# Electrode Size and Placement for Surface EMG Bipolar Detection from the Brachioradialis Muscle: A Scoping Review

**DOI:** 10.3390/s21217322

**Published:** 2021-11-03

**Authors:** Andrea Merlo, Maria Chiara Bò, Isabella Campanini

**Affiliations:** 1 LAM-Motion Analysis Laboratory, S. Sebastiano Hospital, Neuromotor and Rehabilitation Department, Azienda USL-IRCCS di Reggio Emilia, Via Circondaria 29, 42015 Correggio, Italy; andrea.merlo@ausl.re.it; 2 Merlo Bioengineering, 43100 Parma, Italy; chiara.bo@merlobioengineering.com

**Keywords:** brachioradialis, surface electromyography, electrode placement, crosstalk, upper limb

## Abstract

The brachioradialis muscle (BRD) is one of the main elbow flexors and is often assessed by surface electromyography (sEMG) in physiology, clinical, sports, ergonomics, and bioengineering applications. The reliability of the sEMG measurement strongly relies on the characteristics of the detection system used, because of possible crosstalk from the surrounding forearm muscles. We conducted a scoping review of the main databases to explore available guidelines of electrode placement on BRD and to map the electrode configurations used and authors’ awareness on the issues of crosstalk. One hundred and thirty-four studies were included in the review. The crosstalk was mentioned in 29 studies, although two studies only were specifically designed to assess it. One hundred and six studies (79%) did not even address the issue by generically placing the sensors above BRD, usually choosing large disposable ECG electrodes. The analysis of the literature highlights a general lack of awareness on the issues of crosstalk and the need for adequate training in the sEMG field. Three guidelines were found, whose recommendations have been compared and summarized to promote reliability in further studies. In particular, it is crucial to use miniaturized electrodes placed on a specific area over the muscle, especially when BRD activity is recorded for clinical applications.

## 1. Introduction

Surface electromyography (sEMG) is an important tool for monitoring muscle activity but there is still limited teaching about its proper detection and its applications. This is likely the cause of the great variability of detection modalities already pointed out by the SENIAM recommendations over 20 years ago [1]. This work focuses on the brachioradialis muscle (BRD), which is one of the main elbow flexors along with the biceps brachii and the brachialis. It is the only muscle that allows elbow flexion with the forearm placed in a neutral position [2]. In neurological patients, BRD is often affected by structure and muscle activity alterations, leading to pathological upper limb patterns of movement, and therefore is one of the main targets of corrective interventions, such as functional surgery [3].

Surface EMG of elbow flexors is a critical non-invasive assessment technique clinically employed for the definition of the surgical intervention [4] and for the selection of muscles to be treated by focal muscle inhibition [5], complementary to clinical examination. Moreover, BRD is also used in the control of sEMG-guided forearm prostheses [6,7,8,9].

While BRD originates in the arm, running near the biceps brachii and the brachialis, its muscle belly is located at the forearm where it is adjacent to the distal muscles of the wrist and fingers, such as the extensors carpi radialis, the flexors carpi radialis, and the extensor digitorum. Given their anatomical proximity with BRD and the small BRD cross-sectional area, the risk of crosstalk from these muscle on BRD is quite high [10]. Recording an unreliable tracing may compromise the quality of scientific studies on muscle physiology and motor control, where the presence, timing or amplitude of muscle activity are used to answer the research question. Moreover, in clinical applications, where bipolar sEMG assessments of muscle activity are used to plan neuro-orthopedic surgery or to select the muscles to be treated (e.g., by focal inhibition), the presence of crosstalk may lead to even serious errors and must therefore be avoided [11].

However, the issue of crosstalk on BRD has been scarcely investigated in the literature [12]. Even worse, no standardized guidelines for surface electrodes placement on BRD are available and even the SENIAM Recommendations [1] do not provide specific indications for this muscle. It is therefore surprising that many works on BRD refer to the SENIAM project. Despite this lack of standardization, more than a hundred papers on BRD bipolar sEMG have been published in the last two decades. We presume that a lack of awareness exists among researchers with regards to crosstalk and, more generally, on the concept of detection volume in sEMG.

In this study, we conducted a scoping review on the studies assessing BRD muscle activity with bipolar sEMG to identify the electrode configurations used by authors in terms of sensor size, conductive area, interelectrode (IED) distance, reference bibliography (if any), and crosstalk discussion. Moreover, results from the studies aiming at identifying the best electrode configuration using bipolar sEMG have been collected and compared to provide guidelines for reliable measurement of BRD activity in clinical applications. These guidelines apply to most other muscles as well.

## 2. Materials and Methods

The methodology used in this scoping review was described in [13] and consists of six stages, as described below.

### 2.1. Stage 1: Identifying the Research Question

The main questions driving our investigation were: (i) Are there guidelines in the literature that are shared by clinicians for electrode placement? (ii) How are sEMG electrodes commonly placed to register BRD activity? (iii) Is crosstalk with adjacent muscles relevant? (iv) How is crosstalk handled among studies?

### 2.2. Stage 2: Identifying Relevant Studies

Systematic searches were designed, refined, and conducted in September 2021. Medline, Pedro, and Cochrane databases were investigated, and no time limitations were imposed. Only studies in English were included, based on the authors’ language skills.

Keywords employed in the research included “brachioradialis”, “electromyography”, “surface”, “dynamic”, “bipolar”, “differential”. To ensure consistency in terms of this search, Medical Subjects Headings (MeSH) were included when available.

Additional papers not retrieved by the electronic search were then added by hand searching the reference sections of the included studies.

### 2.3. Stage 3: Selecting Studies

*Eligibility criteria*: Selected studies had to include the assessment of BRD muscular activity through the employment of sEMG by bipolar electrodes.

Studies were excluded when dealing with indwelling EMG and high-density surface EMG (HDsEMG) without a simultaneous acquisition of BRD muscular activity with bipolar electrodes. Studies assessing patients after BRD surgical transfer or splinting were also excluded, due to anatomy and function modifications.

*Types of studies*: All primary study types were included in the current scoping review. Both papers and congress abstracts were included.

*Context*: No limitations were imposed regarding the context of the studies.

*Screening*: Two reviewers independently screened all articles and assessed them according to the eligibility criteria. Any discrepancy was discussed, and a third reviewer intervened if necessary. When full text was unavailable, authors were contacted via email and the study was excluded only if they did not reply.

### 2.4. Stage 4: Charting the Data

Relevant data were extracted from each study and collected in specific databases. Extractions were discussed when in doubt and table headings were updated to increase accuracy.

### 2.5. Stage 5: Summarizing and Reporting the Data

Descriptive tables were designed to best summarize relevant data. Findings were presented in a narrative synthesis.

### 2.6. Stage 6: Consultation

While setting inclusion criteria and preparing tables, both bioengineers and clinical experts were contacted to increase the quality of the review and to avoid missing relevant papers. Findings were discussed again at the end of the writing.

### 2.7. Sources of Crosstalk and Glossary of Terms Used in This Review

For the sake of clarity, we report in Table 1 the main terms and concepts addressed in this review, along with their effect on the reliability of sEMG data.

## 3. Results

A total of 237 papers were identified by electronic search. After removing the duplicates, 225 studies were retrieved and 153 were selected for the full-text assessment. Studies were excluded at this stage mainly because they did not assess the BRD activity among the other elbow flexors or because authors employed other types of EMG such as HDsEMG or fine wire EMG.

From these, 130 articles were finally included in the current review to which we added 4 studies retrieved by manual search, leading to a total of 134 studies.

**Figure 1:**
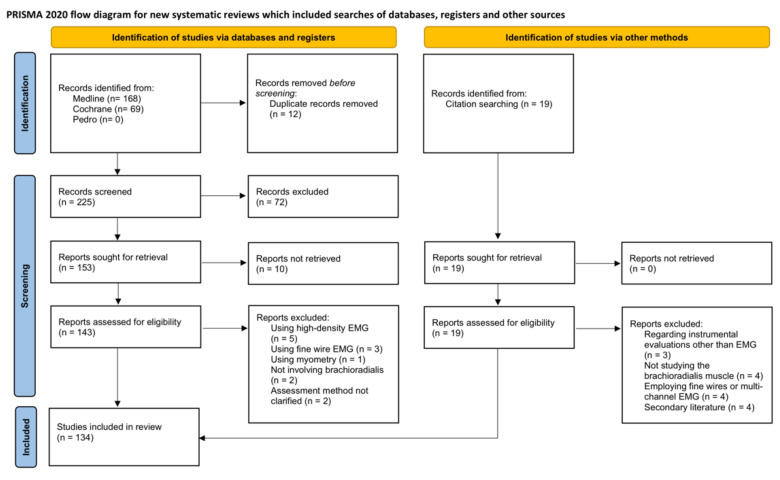
Flow chart of the literature search on surface electromyography of the brachioradialis muscle.

The Flow Chart of the research follows the PRISMA guidelines [15] and is depicted in Figure 1.

### 3.1. Sensor Location Guidelines on the Brachioradials Muscle Available in Literature

Three different guidelines on electrode placement over BRD were followed by a subset of the papers included in this review, as reported in Table 2. Two of these guidelines (Basmajian’s and Barbero) were based on experimental measurements of BRD activity.

In Basmajian’s guidelines, the minimum crosstalk areas for superficial muscles, including BRD, were reported [16]. These areas were identified by covering the muscle belly with a series of small electrodes (Beckman type) and recording their bipolar sEMG signals during a sequence of exercises selected to separately activate the target muscle alone and then the surrounding muscles excluding the targeted muscle. An example of this technique can be found in the paper by Blanc and colleagues [17].

The guidelines by Barbero and colleagues [14] provide instructions based on the 95% confidence interval of the location of the innervation zone (IZ) location, which should be avoided in sEMG acquisitions, as experimentally observed in superficial muscles parallel to the skin in a sample of 20 male and 20 female healthy subjects [14].

The third guidelines, proposed by Cram, provides instructions on how to properly locate the muscle belly and indicate to place the electrodes 4 cm distally from the lateral epicondyle and spaced by 2 cm [10], even though with this configuration “signals from extensor carpi radialis (longus and brevis) and brachioradialis are common” [10].

### 3.2. Studies Assessing the Brachioradialis Muscle by Means of sEMG

A complete list of all 134 studies included in this review is reported in Supplementary Table S1. This includes details starting with: author, year of publication, electrode placement, “electrode” (sEMG sensor external) size, electrode sensitive area, center-to-center distance, application (e.g., physiology, ergonomics, etc.) and assessed subjects (e.g., healthy adults or patients). In addition, papers where crosstalk was assessed or mentioned are highlighted.

For sake of completeness, we reported in Supplementary Table S2 the studies retrieved during our search that used HDsEMG to assess BRD [9,18,19,20,21,22] (See Figure 1). These were excluded from the current review that is focused on bipolar detection.

Table 3 provides a summary of this information, with the studies grouped by type of electrode placement. As many as 80 studies were conducted with the electrodes generically placed over the “muscle belly” or did not report any information on electrode location. SENIAM recommendations [1] were cited by 16 authors, even though no indication on electrode placement over BRD are provided in these guidelines. Similarly, the text by Delagi and Perotto [23] has been cited by six authors even if these guidelines provide instructions for indwelling EMG. The motor point location has been used as a reference by other six authors. The motor point of a muscle is the location of the point with the lowest stimulation threshold and is usually not too far from the IZ. Available guidelines for surface electrode size and location on BRD, reported in Table 2, were followed in six studies only.

Three types of electrodes were used to assess sEMG from BRD: disposable ECG electrodes, non-disposable miniaturized electrodes, and bar electrodes. Snap-button electrodes for ECG applications were the most common choice. Their size ranged from about 10 mm in diameter up to 50 mm, a size that prevents any possibility of placing electrodes on specific locations. Miniaturized sensors with an external diameter of 5 mm or less and a central conductive area varying from 1 to 2.5 mm in diameter were also used and placed on the skin with center-to-center distances ranging from 6 mm up to 30 mm, despite of their small size (see Supplementary Table S1). Bar electrodes, consisting of two rectangular bars of Ag/AgCl, typically 10 × 1 mm in size, spaced by 10 mm and embedded in a plastic or resin probe, were also used.

Most of the studies (78/134) provided information about the BRD physiology in healthy subjects. The ergonomics during different tasks, particularly in surgeons during surgeries, was investigated in seven papers, while 13 authors explored the activity of the BRD while doing sports. Eleven studies focused on the bioengineering field of the signal processing and five on the modelling. Six authors studied sEMG repeatability or signal stationarity and two specifically focused on the crosstalk assessment between BRD and other muscles. When dealing with patients, mostly neurologic, seven studies explored the BRD pathophysiology and only five assessed the BRD activity for clinical purposes.

### 3.3. Studies Mentioning Crosstalk

The term crosstalk appears in 29 of the 134 studies included in this review. In eleven cases it was just cited either in the introduction or in the study’s limitations as a possible confounding factor [8,53,61,78,84,101,122,124,133,143,145]. Two authors assumed that their electrode placement would minimize crosstalk from the biceps brachii [124] and from the forearm muscles [69], and no further explanation was given. Some authors reported that “Every effort was made to control for the limiting factors […] crosstalk” [73] or that electrode placement characteristics “were critically considered to minimize cross-talk between muscles” [151], while no evidence sustaining these claims can be found in their methodological choices. Other authors cited the recommendations on electrode placement reported in Basmajian’s book *Muscle Alive*—a milestone in EMG history—but did not follow them and used large electrodes and relatively large IEDs [97,135].

Three studies [55,126,131] indicated that BRD data were partially affected by crosstalk because of a limited cross-correlation with signals detected on the other muscle assessed in the study (not necessarily in the forearm).

The study by Staudemann and Colleagues [2] aimed at examining whether the activity of the brachialis muscle can be assessed by means of surface EMG without interference from the biceps brachii when using miniaturized electrodes. In this study, the BRD activity was also assessed. The EMG envelope of BRD during an isometric task of flexion with supination at 20% of the maximal force appeared largely different from and unrelated to that of the biceps, thus suggesting (qualitatively) the absence of crosstalk between these two muscles.

### 3.4. Studies Assessing Crosstalk on the Brachioradialis Muscle

Two studies were specifically designed to assess crosstalk on BRD coming from forearm muscles [121,153].

In the study by Mogk and Colleagues [121], the cross-correlation function was used to determine the amount of common signal among seven electrode pairs placed circumferentially around the proximal side of the forearm in six healthy individuals. Pinch and grip tasks were analyzed, with the forearm supported, in three different forearm positions (full pronation, neutral and full supination). Disposable surface electrode pairs (recording surface: 10 mm diameter, area 79 mm^2^, IED 25 mm) were used. These dimensions are typical for pediatric ECG electrodes. Crosstalk was assessed by computing the cross-correlation between couples of sEMG sensors. Unfortunately, authors did not report the results for specific couples of sensors on adjacent muscles (e.g., BRD vs. flexor carpi radialis) but only the average values among all sensors spaced by 3 cm. The mean peak cross-correlation found was in the order of 0.6, thus suggesting a large amount of common signal. This may be due to either crosstalk or to a common drive between motor units, or to a combination of the two. This study indicates that a relatively large amount of crosstalk may exist between adjacent forearm muscles, when sEMG is detected with electrodes of 10 mm in diameter spaced by 25 mm.

In the study by Merlo and Colleagues [153], intramuscular and surface EMG were collected simultaneously and both traces were displayed on a screen in front of the subjects to provide them with online feedback. A support was used for the forearm to prevent the need of BRD activation. Subjects were asked to perform a sequence of wrist flexions and extensions. Intramuscular EMG acquisition was performed by means of fine wires inserted in the BRD according to the recommendations by Perotto [23], under ultrasound guidance and then checked through electrostimulation. Surface EMG signals from three consecutive sessions with different surface electrode configurations were collected. The setups included: (1) a pair of sEMG sensors of 18 mm in external diameter, 78.5 mm^2^ in detection area (10 mm diameter), at 50 mm interelectrode distance (IED) (ARBO H124 SG); (2) a pair of the same sensors placed at 25 mm IED (i.e., tangent to each other); (3) a pair of electrodes of 2.5 mm in diameter of the sensitive area and 5 mm IED (Gereonics Miniature). The presence of activation bursts, with root mean square amplitude at least twice as large as the background noise, in the surface EMG signals with no activity in the fine wire signal (baseline noise only) was classified as crosstalk. Results of this study are summarized in Table 4. Crosstalk almost always occurred when large (ECG) electrodes were used. The authors concluded that disposable snap electrodes typically used for gait analysis do not provide adequate spatial selection when used in the measurement of BRD activity and must be avoided. Miniaturized electrodes can better measure true BRD activity.

## 4. Discussion

In this study our aim was to map the methods used in literature for BRD electrode placement when performing sEMG. We focused on bipolar electrodes because they are employed in everyday clinical activities in most of motion analysis laboratories.

The main finding of this scoping review is the lack of awareness of many researchers, clinicians and reviewers, of the importance of electrodes size and placement in sEMG, even though clear indications are accessible in the literature since the 80s. Small electrodes and narrow spacing must be employed (both generally as well as with small muscles in particular) if high discrimination against crosstalk from adjacent muscles is desired [16,155]. In addition, large electrodes and large IEDs introduce strong filtering that makes comparison of results impossible among publications [156,157].

As many as 106 studies out of 134 (79%) did not even addressed the issue by placing the electrode pair over the muscle belly, or did not report any information on the placement, or referred to guidelines that do not provide recommendation for the BRD muscle or for surface EMG at all (See Table 3). Being “above” the muscle was the main criterion found in literature for electrode placement over BRD. The identification of the motor point used by some authors for selecting the electrode location, which is both a time-consuming technique and a usually wrong approach in sEMG applications–supports our assumption as to the lack of awareness concerning this topic. The motor point of a muscle is the point with the lowest stimulation threshold and is usually not far from the IZ. As the most common electrode montage is differential, if the IZ falls underneath the electrode pair, the resulting signal will be very small and noisy and highly affected by small electrode displacements [14].

About one third of the studies did not provide information on electrode size or sensitive area; most of them indicated only the model’s name and the manufacturer. ECG electrodes were mainly used, and these were greater (often much greater) than 10 mm in diameter with large center-to-center IED (>15 mm). Miniaturized electrodes or short arrays with 10 × 1 mm contact bars have been used in 29 studies only.

In some cases, manufacturers provide sEMG sensor with incorporated snap-buttons or electrode carriers that prevent the use of small size contacts. Although home-made modifications have been reported [17], the realization of specific sensors for sEMG, allowing the use of the probes on the market, is highly desirable also for use on muscles with small section as the muscles of the forearm, the peroneal muscles in the leg and the muscles of children.

The term “electrode” is used with different meanings among authors, either referring specifically to the conductive area only or to the size of the whole sensor, including the external adhesive border. The former is correct and should be encouraged in future studies. The latter, which derives from the common language, should be replaced with the term sEMG sensor, according to SENIAM recommendations [1].

The center-to-center IED generally was a consequence of the size of the sensors, as they can be placed no closer than tangent or minimally superimposed. Hence, the wider the sEMG sensor, the greater the distance in-between and, consequently, the detection volume, the amount of crosstalk (See Table 1), and the filtering.

The effect of a large electrode size and IED can be appreciated in Table 4, where crosstalk from forearm muscles was almost ever found on BRD when IED ≥ 25 mm. The effect of IED on signal filtering can also be appreciated from Figs. 11 to 14 of the open access tutorial by Merletti and Muceli [157]. The results reported in Table 4 are in line with those reported by Cram [10], where mutual crosstalk between extensor carpi radialis and BRD was found when a 20 mm IED was used. This analysis of literature confirms the theoretical indications that sEMG electrodes much smaller than the snap-on ECG electrodes (diameter not greater than 3–5 mm and IED not greater than 8–10 mm) should be used to increase selectivity (therefore reducing amplitude) and consequently decreasing crosstalk from nearby muscles in sEMG acquisition [156].

The presence of crosstalk may result in unreliable clinical information in terms of muscle activation (BRD may result active while it is not) and muscle timing [11].

A certain “misuse” in the citation of the SENIAM recommendations [1] results from this scoping review, given that they do not provide any indication for the BRD muscle (See Table 3). Some authors also claimed adherence to the SENIAM recommendations (IED ≤ 20 mm, now obsolete) but placed their electrodes with IED as large as 36 mm or 50 mm [105,117]. Besides the crosstalk, if the diameter of conductive area of the electrodes is greater than the width of the target muscle, the activity of the nearby muscle covered by the electrode is measured directly and is not possible to separate it, with a bipolar detection, from that of the muscle of interest. Similar considerations apply for the book by Delagi and Perotto [23] (See Table 3), which is a reference for indwelling EMG only, and provides instructions on how to reach a muscle with a needle, e.g., avoiding nerves and vessels.

Manuscripts should always provide geometrical data concerning size, distance, and location of electrodes to allow both repeatability of experiments and comparison of results. Moreover, we would like to encourage reviewers to always check the congruence between the reference cited and the protocol actually used in the study, as it is fundamental for sEMG data reliability.

### 4.1. The Issue of Crosstalk

While the anatomy and function of BRD are well known among authors, the origin and the diffusion of the action potential electric field through a finite, anisotropic, and non-homogeneous conductive volume (resulting in a sEMG affected by inhomogeneities and by the detection system, that is the physics of sEMG) are not. This results in the cultural and educational barriers described in the open-access book [158]. This situation has an impact not only on clinical applications but also where the sEMG is processed to control external devices (prosthesis, exoskeletons, other devices).

In a recent review, Talib and colleagues presented a list of techniques to minimize the risk of crosstalk when recording surface electromyographic signals, including a reduction of IED and electrode diameters, and the simultaneous use of sEMG and fine wire EMG [159].

The use of mathematical models for teaching sEMG is very effective in explaining general concepts, describing the effect of the main geometrical, anatomical and physiological factors, and testing algorithms and filters [160]. However, as specified by [12], techniques that are “....optimal in terms of crosstalk reduction largely depend on anatomy and specifically on fat thickness and skin conductivity. This introduces limitations in the application of models in designing optimal spatial filters.”. Methods are being developed to select the optimal detection technique on the basis of the specific muscle being considered and the related recommendations should be soon revised and updated [161]. Currently, the best indications on optimal electrode placement are obtained through direct measurements during the execution of selective tasks. The simultaneous measurement of the target muscle—whose electric silence is expected—with surface and fine wire electrodes and of selectively recruited surrounding muscles allows for the finding of the areas with no or minimal crosstalk. The use of HDsEMG sensors (e.g., electrode grids) can be of great use in this application. The recording of monopolar signals using a HDsEMG grid with IED ≤ 5 mm provides great flexibility for a-posteriori application and testing of different spatial filters. A few monopolar signals can be linearly combined to obtain SD or DD or Laplacian or other filter configurations, with IED multiple of the IED of the grid [162].

### 4.2. Reccomendation on Electrode Placement on the Brachioradialis Muscle

Theoretical considerations and experimental findings indicate that the main criteria to follow for electrode choice and positioning on the BRD muscle are those proposed by Basmajian [16] and Barbero [14]. In summary, electrodes should be small (diam. < 3–5 mm), close to each other (IED < 8–10 mm), slightly distal with respect to the IZ, which is in the proximal third of the muscle, and aligned with the fiber direction along the muscle longitudinal midline. These indications applies when bipolar sEMG is used to measure presence, timing, amplitude, and morphology of voluntary or reflex muscle activation, as typically done in clinical applications and movement analysis [11]. The proposed placement may or may not be optimal for measuring evoked potentials as in the case of electrical or magnetic stimulation [163].

### 4.3. Implications for Clinical Practice

The assumption that underlies the clinical use of the sEMG signal is that the recorded signal derives only from the target muscle, without being corrupted by signals from the neighboring synergistic or antagonistic muscles. That is, the signal must not contain crosstalk. In addition, the signal should not display fictitious amplitude modulations, that could occur if the IZ slides under the electrodes while performing tasks. The signal itself does not contain information on the presence of these two phenomena because there are no current signal processing techniques able to identify the presence of crosstalk from the trace recorded from a single muscle. An aesthetically pleasing signal with recruitment and de-recruitment is very simple to obtain with sEMG. However, only close attention and precision in using the validated placements of small and near electrodes, together with tests requiring neighboring muscles to activate without the target muscle, can ensure a reliable signal. An example of the effects of 1 cm electrode misplacement on sEMG recorded from leg muscles during walking was illustrated in [164].

As it is possible to obtain reliable sEMG acquisition for clinical applications, and this provide valuable information that can improve the treatment appropriateness [165,166,167,168], we strongly encourage professionals to follow the guidelines, especially when the signal is used to make clinical decisions, such as when planning neuro-orthopedic surgery or focal muscle inhibitions [169]. The smaller the cross-sectional area of the muscles of interest (e.g., forearm muscles, children’s muscles), the more rigor must be used.

### 4.4. Available Tutorials and Teaching Material on sEMG

The results of this scoping review outline that researchers, biomedical engineers, and clinicians, as well as many reviewers, do not seem to attach a great deal of importance to the issue of electrode size and placement, as long as one detects or sees a reasonably clean signal. They do not seem to be aware of the fact that the geometrical properties of the detection system affect the signal and its features that may later be used to draw conclusions and make decisions. This point impacts on the quality and reliability of publications and reports and on clinical decisions.

Several on-line tutorials and free teaching material have been developed in the last decade to promote accuracy in sEMG acquisitions [160,170]. Educational initiatives of Scientific Societies and Journals may be very useful [171].

We believe that the detection and interpretation of sEMG signal should be taught at the academic level, as is done for other biosignals (e.g., ECG and EEG), and that international protocols should be developed and approved to reduce this barrier to the widespread clinical use of sEMG. As outlined in the recent literature, there is an urgent need for better education of healthcare personnel in sEMG and other medical technologies [152,153,163,167].

## 5. Conclusions

Analyzing available literature highlights that the issue of crosstalk is little known among researchers and is therefore neglected. This negatively affects the quality and reliability of the studies conducted and can have serious consequences in clinical practice when using sEMG. To overcome this lack of awareness, appropriate information should be taught at university and ongoing training is essential to create a common culture about crosstalk. 

The analysis and synthesis of available literature provides guidelines for the best placement of surface electrodes when assessing the BRD muscle activity. The use of miniaturized electrodes is of paramount importance, with dimensions much smaller than those of button electrodes for electrocardiography placed distally to the IZ that are usually employed in motion analysis laboratories. Reviewers should carefully scrutinize papers and control if the choice of electrode geometry is justified by clearly explained reasons.

When BRD activity is recorded for clinical applications, it is essential to use miniaturized electrodes properly placed according to the guidelines or to use fine-wire EMG.

## Figures and Tables

**Table 1:** Glossary of terms used in this review.

Term	Definition	Effect on sEMG Signal
sEMG sensor ^1^ (often confused with the commercial term “electrode”)	System carrying and including the electrode(s) and their fixation system (e.g., adhesive rings)	Adhesive disks or straps might limit skin elasticity and create artifacts due to micromovements.
Electrode (or electrode sensitive area)	Conductive surface in contact with the skin (dry or wet, e.g., gel)	The voltage distribution on the skin under the electrode takes a single instantaneous value over the entire electrode (average in space). causing lowpass filtering.
sEMG sensor diameter or size	Diameter or size of the whole sensor applied over the skin	Large sEMG sensors require wide inter-electrode distance
Center to center inter-electrode distance (IED)	Distance between electrode centers	Larger IED results in larger detection volume and larger sEMG signal, which is often incorrectly considered a good thing, with the risk of crosstalk
Detection volume	Volume and shape of the region of 3D space containing motor units whose potential can be detected	Region containing motor units whose potentials are above the noise level.
Crosstalk	Signal detected on the target muscle but generated by the motor units of another muscle	When nearby muscles are active the muscle of interest seems to be active, leading to wrong conclusions/decisions.Crosstalk may critically affect clinical decision making
Innervation zone (IZ)	Physical region where the central (alpha-motor neuron terminations) and peripheral (muscle fibers) systems connect through special synapses [14]	During dynamic contractions, the relative movement of the muscle with respect to the skin (that is the electrode system) determines a strong alteration (e.g., reduction) of the signal amplitude when the IZ shifts under the electrode pair [14]

^1^ As defined in the SENIAM recommendations.

**Table 2:** Reference guidelines available in literature for sensor location on the brachioradialis muscle, when assessed by bipolar sEMG.

Author, Year	Criterion	Sensor Location
Basmajian, 1983 [16]	Minimum crosstalk area, determined experimentally	“With the hand pronated and the elbow bent, draw a line from the ¾ point of the elbow skin crease to the styloid process of the radius (in the snuff-box). Place both electrodes centered in an oval area approximately 25–30% the distance from the elbow skin crease to the styloid process of the radius”. This placement refers exclusively to miniaturized electrodes, i.e., with a diameter of a few millimeters, placed next to each other or with a minimum center-to-center distance.
Cram, 1998 [10]	Muscle anatomy	“Palpate the muscle mass just distal to the elbow while resisting elbow flexion with the wrist in the neutral position (thumb up). Two active electrodes, 2 cm apart, are placed approximately 4 cm distally from the lateral epicondyle of the elbow on the medial fleshy mass that covers that area, so that they run parallel to the muscle fibers.”
Barbero, 2012 [14]	Away from the innervation zone	With the elbow extended, draw “a line from the styloid process to a midpoint on the line between the lateral and medial epicondyles. Optimal electrode site: Between 32% and 100% of this line”, starting from the epicondyle (i.e., avoid the very proximal part of the muscle belly, where the innervation zone is most likely located). No indication on electrode size is provided but small electrodes are assumed.

**Table 3:** Electrode configuration and characteristics of the studies assessing brachioradialis muscle activity by bipolar sEMG, as reported in the studies included in this review.

Electrode Location Indicated by Authors	Number of Studies	Reference Numbers	Electrode or sEMG Sensor Diameter or Size, mm	Center to Center Inter-Electrode Distance, mm	Crosstalk Mentioned, No. of Studies	Applications (no. of Studies)	Subjects Assessed (No. of Studies)
Muscle mid-belly ^1^	55	[24,25,26,27,28,29,30,31,32,33,34,35,36,37,38,39,40,41,42,43,44,45,46,47,48,49,50,51,52,53,54,55,56,57,58,59,60,61,62,63,64,65,66,67,68,69,70,71,72,73,74,75,76,77,78]	2–35, or 10 × 3 bars	6–50	7	physiology (36), sport (6), EMG methodology (3), ergonomics (3), signal processing (3), pathophysiology (2), modelling (2)	healthy adults (51), neurologic adults (3), children with cerebral palsy (1)
Not specified	25	[79,80,81,82,83,84,85,86,87,88,89,90,91,92,93,94,95,96,97,98,99,100,101,102,103]	4–22.5 or 1 × 10 bars	1.7–30	4	physiology (13), signal processing (4), clinical (3), sport (2), pathophysiology (1), ergonomics (1), modeling (1)	healthy adults (21), neurologic adults (4)
SENIAM cited, while SENIAM does not provide indications for BRD	16	[6,8,104,105,106,107,108,109,110,111,112,113,114,115,116,117]	4–40, or 10 × 2 bars	2–50	3	physiology (7), pathophysiology (1), sport (3), signal processing (3), modelling (1), EMG methodology (1)	healthy adults (14), neurologic adults (1), children with cerebral palsy (1)
anatomical locations provided, without references to bibliography-^2^	12	[118,119,120,121,122,123,124,125,126,127,128,129]	8–30, or 10 × 1 bars	10–25	2	physiology (8), ergonomics (1), pathophysiology (1), clinical (1), crosstalk assessment (1)	healthy adults (10), neurologic adults (2)
Motor point based	6	[130,131,132,133,134,135]	4–30	15–20	2	physiology (5), sport (1)	healthy adults (6),
Delagi and Perotto 1974 cited, while it provides indications for indwelling EMG only	5	[136,137,138,139,140]	10–34	20–50	2	physiology (1), pathophysiology (1), EMG methodology (1), sport (1), signal processing (1)	healthy adults (4), orthopedic adults (1)
Figure provided	5	[2,141,142,143,144]	2–na ^3^	12–na	2	physiology (2), ergonomics (1), clinical (1), EMG methodology (1)	healthy adults (4), neurologic adults (1)
Barbero 2012	3	[145,146,147]	4–10	20–30	0	physiology (2), ergonomics (1)	healthy adults (3)
Between innervation zone and terminal tendon	3	[148,149,150]	8–na	20–25	2	physiology (2), pathophysiology (1)	healthy adults (2), neurologic adults (1)
Cram 1992	2	[151,152]	na	20	1	physiology (1), modeling (1)	healthy adults (2)
Basmajian 1983	1	[153]	2.5	10	1	crosstalk assessment (1)	healthy adults (1)
Minimal crosstalk areas experimentally found	1	[154]	1 × 10 bars	10	1	physiology (1)	healthy adults (1)

^1^ Reported as: muscle belly, muscle mid-belly, or similar; ^2^ reported as: 2–5 cm distal to the elbow joint/the anticubital fossa/the elbow crease, 20–30% the distance between the medial/lateral epicondyle and the processus styloideus radii/the radial carpal joint/the distal head of the radius, or similar. ^3^ na: not available.

**Table 4:** Occurrence of crosstalk in the brachioradialis (BRD) muscle during wrist flexion and extension tasks when sEMG is detected, from the BRD, using electrodes of different size and interelectrode distance. Absence of fine wire EMG in the BRD was used as the gold standard to determine the presence of crosstalk in the surface signal.

Task	Electrode Diameter 18 mm, IED 50 mm	Electrode Diameter 18 mm, IED 25 mm	Electrode Diameter 2.5 mm, IED 10 mm
Wrist extension	13/13	13/16	3/15
Wrist flexion	10/14	8/16	1/15
Finger extension	4/5	3/5	1/6
Total	27/32 (84%)	24/35 (69%)	5/36 (14%)

Modified from Merlo 2009 [153] with author’s permission.

## Data Availability

Not applicable.

## Supplementary Materials

The following are available online at https://www.mdpi.com/article/10.3390/s21217322/s1, Table S1: complete list of all studies included in this scoping review. Table S2: List of the retrieved studies using high-density EMG.
