# Supplementary material for: Electrode Size and Placement for Surface EMG Bipolar Detection from the Brachioradialis Muscle: A Scoping Review
Source: Sensors (Basel). 2021 Nov 3;21(21):7322. doi: 10.3390/s21217322 (PMC8587451; doi:10.3390/s21217322)
Supplement: Supplementary file 1 [file sensors-21-07322-s001.zip › sensors-1423382-supplementary.pdf]

Supplementary Table S1 – Complete list of all studies included in the current scoping review.

Author, year	Ref.	Electrode Placement	Electrode or sEMG sensor diameter or size, mm	Electrode conductive area, mm^2	Center to center inter-electrode distance, mm	Crosstalk assessed	Crosstalk mentioned	Application	Subjects
Cavanagh 1979	[79]	na	10	na	na			physiology	healthy adults
Matthews 1980	[75]	over muscle belly	35	na	35			physiology	healthy adults
Matthews 1981	[83]	na	na	na	na		yes	physiology	healthy adults
Tang 1981	[24]	over muscle belly	10	na	50			pathophysiology	patients with hemiplegia
Thepaut-mathieu 1985	[93]	na	na	na	10			physiology	healthy adults
Pinzur 1988	[85]	na	na	na	na			clinic/surgical planning	patients with spasticity
Thepaut-mathieu 1988	[93]	na	4	na	15			physiology	healthy adults
Gamet 1989	[64]	over muscle belly	5	na	20			physiology	healthy adults
Nagata 1990	[37]	over muscle belly	4	na	10			physiology	healthy adults
Rouard 1990	[48]	over muscle belly	11	na	na			sport	healthy adults
Bell 1992	[57]	over muscle belly	na	na	30			physiology	healthy adults
De serres 1992	[32]	over muscle belly	4	na	15			physiology	healthy adults
Krogh-lund 1992	[133]	distal to the motor point	na	18.5	15			physiology	healthy adults
Petitjean1992	[40]	over muscle belly	5	na	20			physiology	healthy adults
Caldwell 1993	[141]	Figure provided	na	3.14	12	yes	yes	physiology	healthy adults
Jamison 1993	[143]	Figure provided	na	3.14	12			physiology	healthy adults
Krogh-lund 1993	[132]	distal to the motor point	na	18.5	15			physiology	healthy adults
Krogh-lund 1993	[131]	distal to the motor point	na	18.5	15		yes	physiology	healthy adults
Nakazawa 1993	[28]	over muscle belly	7	na	20			physiology	healthy adults
Bechtel 1994	[65]	over muscle belly	2	na	10			physiology	healthy adults
Theeuwen 1994	[90]	na	na	na	na		yes	physiology	healthy adults
Rouard 1995	[69]	over muscle belly	11	na	20		yes	sport	healthy adults
Weeks 1996	[154]	minimal crosstalk areas experimentally found	1x10	10	10	yes	yes	physiology	healthy adults

Bilodeau 1997	[52]	over muscle belly	4	na	6			EMG methodology	healthy adults
Tal'nov 1997	[50]	over muscle belly	na	na	na			modeling	healthy adults
Kahn 1998	[101]	na	na	na	na			physiology	healthy adults
Kasprisin 1998	[38]	over muscle belly	na	50	20			EMG methodology	healthy adults
Tal'nov 1999	[47]	over muscle belly	7.5	na	20			physiology	healthy adults
Guevel 2000	[42]	over muscle belly	4	na	13			physiology	healthy adults
Kasprisin 2000	[49]	over muscle belly	na	50	20			physiology	healthy adults
Siemionow 2000	[56]	over muscle belly	na	50	na			physiology	healthy adults
Yamamoto 2000	[29]	over muscle belly	7	na	15			physiology	healthy adults
Levy 2001	[102]	na	na	na	na			physiology	healthy adults
Mamaghani 2001	[82]	na	na	na	na			physiology	healthy adults
Ozawa 2001	[144]	Figure provided	<10	na	>30			ergonomy	healthy adults
Schmit 2001	[45]	over muscle belly	na	na	na			pathophysiology	patients with stroke and brain injury
Mamaghani 2002	[81]	na	na	na	na			physiology	healthy adults
O'Sullivan 2002	[138]	Delagi & Perotto 1974	na	na	20		yes	physiology	healthy adults
Page 2003	[139]	Delagi & Perotto 1974	12	113	20			physiology/clinics	healthy adults and orthopaedic patients
Praagman 2003	[86]	na	na	na	1.7			physiology	healthy adults
Seghers 2003	[110]	SENIAM*	10	na	20			physiology	healthy adults
Tarata 2003	[89]	na	22,5x22,5	506	25		yes	signal processing	healthy adults
Todd 2003	[61]	over muscle belly	10	na	na			physiology	healthy adults
Mogk 2003	[121]	one third of the distance from the proximal end of a line from the medial epicondyle to the distal head of the radius	10	79	25	yes	yes	crosstalk assessment	healthy adults
Barry 2004	[25]	over muscle belly	8	na	20			physiology	healthy adults
Hostens 2004	[111]	SENIAM*	10	na	20			signal processing	healthy adults
Schulte 2004	[41]	over muscle belly	na	16	20			physiology	healthy adults

Barry 2005	[51]	over muscle belly	na	na	13			physiology	healthy adults
Cui 2005	[80]	na	na	na	na			signal processing	healthy adults
Ervilha 2005	[122]	5 cm distally from elbow joint	na	na	20			physiology	healthy adults
Jaskolska 2006	[35]	over muscle belly	4	na	25		yes	physiology	healthy adults
Linnamo 2006	[134]	distal to the motor point	4	na	20			physiology	healthy adults
Søgaard 2006	[62]	over muscle belly	10	na	na			physiology	healthy adults
Neto 2007	[112]	SENIAM*	10x2	20	20			sport	healthy adults
Smith 2007	[59]	over muscle belly	10	na	na			physiology	healthy adults
Calder 2008	[130]	over motor point	10x30	na	20			physiology	healthy adults
Chang 2008	[60]	over muscle belly	10	na	na	yes	yes	signal processing	healthy adults
Doheny 2008	[113]	SENIAM*	1x10	10	10			physiology	healthy adults
Iwamuro 2008	[100]	na	na	na	na			clinics	patients with stroke
Mayer 2008	[124]	4 cm distal to the elbow crease	na	na	na		yes	clinics	patients with acquired brain injury
Rudroff 2008	[148]	between innervation zone and terminal tendon	8	na	20		yes	physiology	healthy adults
Bonnefoy 2009	[114]	SENIAM*	40	na	25			physiology	healthy adults
Hug 2009	[71]	over muscle belly	10x1	10	10			physiology	healthy adults
Li 2009	[151]	Cram 1998	na	na	20		yes	modeling	healthy adults
Oliveira 2009	[126]	5 cm distally from elbow joint	30	78.5	20			physiology	healthy adults
Oliveira 2009	[125]	5 cm distally from elbow joint	30	78.5	20			physiology	healthy adults
Staudenmann 2009	[127]	2 cm distal to the elbow joint	8	na	20		yes	physiology	healthy adults
Merlo 2009	[153]	Basmajian 1983	from 2.5 to 18	na	from 10 to 50	yes	yes	crosstalk assessment	healthy adults
Lopez 2010	[6]	SENIAM*	na	na	na			signal processing	healthy adults
Mountjoy 2010	[70]	over muscle belly	na	na	na	yes	yes	modelling	healthy adults
Gerzevic 2011	[115]	SENIAM*	9	na	20			sport	healthy adults
Hawkes 2011	[97]	na	na	78.5	20		yes	physiology	healthy adults
Booghs 2012	[46]	over muscle belly	8	na	na			physiology	healthy adults

Butler 2012	[53]	over muscle belly	na	na	40			physiology	healthy adults
Hashemi 2012	[116]	SENIAM*	4	na	15			modeling	healthy adults
Holmes 2012	[149]	between innervation zone and terminal tendon	na	na	25			physiology	healthy adults
James 2012	[55]	over muscle belly	na	na	na			physiology	healthy adults
Serrau 2012	[87]	na	na	na	20			sport	healthy adults
Santhome 2012	[117]	SENIAM*	36	78	36			physiology	healthy adults
Uzun 2012	[150]	halfway from the motor point area to the distal insertion	na	na	20		yes	pathophysiology	adults with spinal cord injury
Baudry 2013	[33]	over muscle belly	8	na	20			physiology	healthy adults
Caldiroli 2013	[68]	over muscle belly	10	na	10			ergonomy	healthy adults
Kang 2013	[63]	over muscle belly	25	na	20			physiology	healthy adults
Lampropoulou 2013	[152]	Cram 1998	na	na	na			physiology	healthy adults
Semmler 2013	[31]	over muscle belly	4	na	20			physiology	healthy adults
Yoon 2013	[104]	SENIAM*	8	na	16			physiology	healthy adults
Chalmers 2014	[44]	over muscle belly	na	na	na			physiology	healthy adults
Grant 2014	[58]	over muscle belly	na	na	25		yes	sport	healthy adults
Harwood 2014	[91]	na	na	na	20			physiology	healthy adults
Hong 2014	[98]	na	na	na	na			sport	healthy adults
McCormick 2014	[73]	over muscle belly	na	na	25		yes	sport	healthy adults
Onety 2014	[36]	over muscle belly	10x1	10	10			ergonomy	healthy adults
Rong 2014	[140]	Delagi & Perotto 1974	na	na	50			signal processing	healthy adults
Thiebaud 2014	[128]	at 20% of the distance between the fossa cubit and radial carpal joint	na	na	na			physiology	healthy adults
Carius 2015	[8]	SENIAM*	15	95	20		yes	EMG methodology	healthy adults
Hiscock 2015	[137]	Delagi & Perotto 1974	34	na	30			sport	healthy adults
Rendos 2015	[135]	proximal to the motor point	na	na	na		yes	sport	healthy adults
Sonne 2015	[74]	Over muscle belly	na	na	na			physiology	healthy adults
Staudenmann 2015	[2]	Figure provided	2	na	10	yes	yes	EMG methodology	healthy adults

Vonwerder 2015	[129]	one third from the fossa cubit along the palpable muscle belly	na	na	20	yes	physiology	healthy adults
Z'graggen 2015	[120]	25% of the distance from the lateral epicondyle of the humerus to the styloid process of the radius	8	na	na		physiology	healthy adults
Dickie 2016	[119]	3 cm lateral 4 cm below the anticubital fossa	na	na	20		physiology	healthy adults
Gaudet 2016	[136]	Delagi & Perotto 1974	10	na	24	yes	EMG methodology	healthy adults
Hill 2016	[145]	Barbero 2012	4	na	30		physiology	healty adults
Padulo 2016	[105]	SENIAM*	na	na	50		physiology	healthy adults
Penzer 2016	[84]	na	8	na	20		physiology	healthy adults
Turpin 2016	[67]	over muscle belly	10x1	10	10		physiology	healthy adults
Vonwerder 2016	[106]	SENIAM*	na	na	20	yes	physiology	healthy adults
Baadjou 2017	[118]	at one-fourth of the distance between the elbow crease and processus styloideus radii	10x1	10	10		ergonomy	healthy adults
Issa 2017	[99]	na	na	na	30		physiology/diagnostic	patients with ALS
Ranavolo 2017	[146]	Barbero 2012	10	na	20		ergonomy	healthy adults
Sarcher 2017	[107]	SENIAM*	10	na	na	yes	physiology/clinics	children with CP
Zhang 2017	[95]	na	10x1	10	10		ergonomy	healthy adults
Caufriez 2018	[66]	over muscle belly	na	na	20	yes	physiology	healthy adults
Hill 2018	[147]	Barbero 2012	4	na	30		physiology	healthy adults
Liu 2018	[30]	over muscle belly	10x1	10	10		physiology	patients with stroke
Myers 2018	[78]	over muscle belly	10x1	10	10		physiology	healthy adults
Myers 2018	[26]	over muscle belly	10x1	10	10		physiology	healthy adults
Sarcher 2018	[54]	over muscle belly	21	78.5	22		EMG methodology	children with CP
Sommer 2018	[34]	over muscle belly	4	na	20		signal processing	healthy adults

Zeng 2018	[94]	na	10x1	10	10	signal processing	healthy adults
Zhang 2018	[27]	over muscle belly	10x1	10	10	signal processing	healthy adults
Marcolin 2018	[108]	SENIAM*	na	na	24	sport	healthy adults
Becker 2019	[109]	SENIAM*	na	na	2	signal processing	healthy adults and patients with spasticity
Donges 2019	[142]	Figure provided	na	na	na	clinics	patients with cervical spinal cord injury
Laßek 2019	[123]	one third from the cubital fossa	na	na	20	physiology/diagnostic	healthy adults and patients with spasticity
Myers 2019	[76]	over muscle belly	10x2	10	10	physiology	healthy adults
Myers 2019	[77]	over muscle belly	10x3	10	10	physiology	healthy adults
Suplino 2019	[88]	na	na	na	na	modeling/exoskeletons	healthy adults
Chaytor 2020	[72]	over muscle belly	na	na	20	sport	healthy adults
Hsin-Chien Lee 2020	[96]	na	na	na	na	clinics	patients with stroke
Lv 2020	[103]	na	10x1	10	na	signal processing	healthy adults
Pratt 2020	[43]	over muscle belly	12	113	17	sport	healthy adults
Watanabe 2021	[39]	over muscle belly	na	na	22	ergonomy	healthy adults

* SENIAM cited, while SENIAM does not provide any reference for BRD; na = not available

Supplementary Table S2 –List of the retrieved studies using high-density EMG.

Author, year	Ref.	Electrode placement	High-density EMG	Electrode size	Inter-electrode distance	Crosstalk assessed	Crosstalk mentioned	Application	Subjects
Lowery 2002	[18]	Bars placed such that the long axis of each electrode bar was perpendicular to the muscle fiber direction	Four parallel electrode bars	10x1 mm	8 mm			Physiology	Healthy adults
Lowery 2003	[19]	Bars placed parallel to the fiber direction, and away from the innervation zone of the muscle	Four parallel electrode bars	10x1 mm	8 mm		yes	Physiology	Healthy adults
Gazzoni 2014	[9]	Stretchable sleeve with the columns of electrodes placed around the forearm circumference such as the first column was in correspondence of the ulna	Grid of 112 circular electrodes	6 mm diameter	15 mm		yes	Physiology	Healthy adults
Jafari 2014	[20]	Either proximally or distally from the main IZ location	Linear array of 8 electrodes	na	5 mm			Physiology	Healthy adults
Boccia 2015	[21]	Selected by visual inspection of the sEMG signals	Linear array of 8 electrodes	5x1 mm	5 mm			Sport	Healthy adults
Hajian 2019	[22]	The fourth electrode of the array was placed at one-third the length of the forearm measured from the elbow	Linear array of 8 electrodes	na	5 mm			Physiology	Healthy adults

na = not available
